# Clinical application of exhaled nitric oxide measurement in pediatric lung diseases

**DOI:** 10.1186/1824-7288-38-74

**Published:** 2012-12-31

**Authors:** Angelo Manna, Carlo Caffarelli, Margherita Varini, Carlotta Povesi Dascola, Silvia Montella, Marco Maglione, Francesco Sperlì, Francesca Santamaria

**Affiliations:** 1 Department of Pediatrics, Federico II University, Via Sergio Pansini, 5 80131, Naples, Italy; 2 Department of Pediatrics, University Hospital of Parma, Parma, Italy

**Keywords:** Exhaled nitric oxide, Children, Airway diseases, Asthma, Bronchiolitis, Community acquired pneumonia, Bronchiectasis, Diffuse lung disease

## Abstract

**Summary:**

Fractional exhaled nitric oxide (FeNO) is a non invasive method for assessing the inflammatory status of children with airway disease. Different ways to measure FeNO levels are currently available. The possibility of measuring FeNO levels in an office setting even in young children, and the commercial availability of portable devices, support the routine use of FeNO determination in the daily pediatric practice. Although many confounding factors may affect its measurement, FeNO is now widely used in the management of children with asthma, and seems to provide significantly higher diagnostic accuracy than lung function or bronchial challenge tests. The role of FeNO in airway infection (e.g. viral bronchiolitis and common acquired pneumonia), in bronchiectasis, or in cases with diffuse lung disease is less clear. This review focuses on the most recent advances and the current clinical applications of FeNO measurement in pediatric lung disease.

## Introduction

Nitric oxide (NO) is a biological mediator synthesized by NO synthase (NOS), an enzyme that catalyzes the oxidation of L-arginine to NO and L-citrulline. Constitutive NOS isoenzymes include neuronal NOS (NOS1) and endothelial NOS (NOS3), both of which are activated by calcium ions to produce small amounts of NO [1]. Inducible NOS (NOS2), that is induced by inflammatory and infectious stimuli, produces large amounts of NO independent of calcium ion influx [2]. NO was first described as a vascular smooth muscle relaxant and was subsequently found to be present in the expired breath of animals and humans [1]. In the lungs, NO determines smooth muscle relaxation, affects ciliary beat frequency, mucus secretion and plasma exudation, and is involved in neurotransmission, blood flow regulation, inflammation and cell-mediated immunity processes [3]. In the upper respiratory tract NO levels are higher than in the lower airways, with the maximal concentration in the paranasal sinuses, probably because of local increased NOS activity and of poor ventilation [4].

During the last years the availability of NO analyzers and the publication of official guidelines made the measurement of fractional exhaled NO (FeNO) a useful tool in the diagnosis of various pediatric airway disorders [5-10]. This review summarizes the most recent advances and the current clinical applications of FeNO measurement in the pediatric clinical practice.

### How should FeNO be measured?

Several methods are used to measure FeNO in children, and the choice depends on the subject’s age and cooperation. Online measurement allows FeNO testing with a real-time display of NO breath profiles, which is available in stationary devices only. Offline testing, instead, refers to collection of exhaled air into suitable receptacles for delayed analysis [5]. The single breath online measurement is the gold standard in school-age children [5,11]. The subject is asked to inhale to near total lung capacity and to exhale at a constant flow of 50 mL/s until a NO plateau of at least 2 seconds can be identified. The offline method with constant flow rate is the offline method of choice in school-age children [5]. The child blows air through a mouthpiece into a receptacle. Nasal contamination is prevented by closing the velum by exhaling against at least 5 cm H_2_O oral pressure. NO concentrations in balloons can be stable for several hours, and the measurement can take place also at a distant site. Flow rate standardization improves the reproducibility of the offline technique, with results similar to the online constant flow rate methods [11]. In subjects younger than 5 years, single-breath online measurement is not well standardized since children cannot adequately cooperate. In the age group from 2 to 5 years, FeNO levels are measured online during spontaneous breathing, with the exhalation flow adjusted to 50 mL/s by changing the exhalation resistance [12]. The child breathes quietly, slowly and regularly through a mouthpiece connected to a two-way valve. NO-free air is continuously flushed through the inlet of the valve. However, NO levels measured during spontaneous breathing may not equate with single-breath online measurements. In children younger than 2 years, the tidal breathing method has been used both online and offline, but it is not standardized [11].

Finally, the agreement between different devices gave inconsistent results, with some studies showing significant differences between analyzers [13], while others showed a high degree of agreement between different devices [14-16]. In particular, a recent study demonstrated an acceptable degree of agreement only between devices from the same manufacturer, both stationary and portable [17].

### FeNO reference values in exhaled air

Several recent publications have reported reference values for FeNO in children and adolescents [18-28] (Table 1). There are important differences among these studies regarding to the size of the examined population as well as the range of statistical variables that have been included or excluded, limiting their value. Several factors may affect FeNO levels: measurement techniques, exhalation flow rate, nasal NO contamination, NO analyzer used, race, age, sex, atopy, anthropometric measures, smoking, and diet. While some of these (height, age, smoke, atopy) are universally accepted as crucial in increasing FeNO values [20,29], there is uncertainty regarding the others [30-32].

**Table 1:** FeNO reference values in healthy children and adolescents

**Author [reference]**	**N**	**Subjects**	Normal values*	**Analyzer**
**Dötsch 1996 **[24]	37	Caucasian, 4-18 years	3.1 ppb	CLD 700; Eco Physics, Dürnten, Switzerland
**Baraldi 1999**[18]	159	Caucasian, 6–15 years	8.7 ppb	CLD 700, Al-Med, Ecophysics, Durnten, Switzerland
**Silvestri 1999**[25]	22	Caucasian, 11-12 years	4.0 ppb	LR 2000, Logan System, Rochester, Kent, UK
**Franklin 1999**[26]	157	Caucasian, 7-13 years	7.4 ppb	NOA 280, Seivers Instruments Inc., Boulder, CO
**Latzin 2002**[27]	107	Caucasian, 4-18 years	9.2 ppb	LR 2000, Logan Research, Rochester, UK
**Jouaville 2003**[28]	96	Caucasian, 9-10 years	13.3 ppb	Sievers Instruments, Boulder, CO, USA, kit bag collection
**Buchvald 2005**[19]	405	Caucasian, Hispanic, Asian, Black, 4–17 years	9.7 ppb	NIOX, Aerocrine AB, Stockholm, Sweden
**Wong 2005**[21]	291	Chinese, 11-18 years	19.9 ppb	NIOX, Aerocrine AB, Stockholm, Sweden
		Caucasian, 11-18 years	12.7 ppb	
**Malmberg 2006**[22]	114	Caucasian, 7.2-15.7 years	Mean values not reported; range 7-14 ppb	NIOX, Aerocrine AB, Stockholm, Sweden
**Kovesi 2008**[20]	657	White, Black, Asian, 9.1-12.9 years	12.7 ppb	CLD 88sp, Eco Medics AG; Durnten, Switzerland
**Yao 2012**[23]	693	Asiatic, 5-18 years	13.7 ppb	CLD 88sp, Eco Medics AG; Durnten, Switzerland

*Expressed as mean values.

### FeNO and bronchial asthma

#### FeNO in the diagnosis of bronchial asthma

Chronic airway inflammation is an important feature in the development and progression of bronchial asthma [10,33-36]. FeNO levels are increased in asthmatic patients [37-39] as a result of induction of NOS2 by proinflammatory cytokines [3,40-43]. There is some degree of correlation between elevated FeNO levels and increased eosinophils in blood, bronchoalveolar lavage fluid [28], bronchial biopsies [44], sputum [45], this indicating that FeNO reflects eosinophilic inflammation [28,33,46,47]. However, asthma is probably not a single disease since at least 3 adult phenotypes of airway inflammation have been identified on the basis of predominant eosinophilic, neutrophilic, or pauci-granulocitic cellular pattern [48]. It is therefore not surprising that the difference in FeNO levels between symptomatic and asymptomatic children is relatively small, and a large overlap in the distribution of FeNO levels from subjects with and without asthma has been reported [49-58] (Table 2). Another limitation is that even though FeNO is increased in children with allergic asthma [59-62], the obtained levels cannot discriminate among schoolchildren with non-allergic asthma, or those with allergy without asthma, or the healthy population [63]. Moreover, levels of FeNO appear increased in asthmatics with pollen allergy during the season also in the absence of symptoms of lung function impairment [64]. Children with atopic eczema exhibit high levels of FeNO even though they do not have asthma [65], but the mechanism is unclear.

**Table 2:** Diagnostic accuracy of FeNO measurement in patients with asthma

**Author [reference]**	**Subjects**	**Patients with asthma (%)**	**FeNO cut-point**	**Sensitivity (%)**	**Specificity (%)**
**Malmberg 2003**[58]	Children, 3-7 years	83 (25)	> 9.7 ppb	86	92
**Dupont 2003**[50]	Adults	160 (66)	> 16 ppb	49	90
**Berkman 2005**[57]	Adults	40 (47)	> 7 ppb	82	89
**Sivan 2009**[51]	Children, 12-18 years	106 (70)	> 19 ppb	86	89
**Sachs-Olsen 2010**[52]	Children, 10-12 years	60 (45)	> 20.4 ppb	41	97
**Pérez Tarazona 2011**[53]	Children, 6-14 years	57 (40)	> 19 ppb	91	87
**Yao 2011**[54]	Children, 5-18 years	70 (4.5)	> 28 ppb	64	70
**Cordeiro 2011**[55]	Adults, Children	42 (30)	> 27 ppb	78	92
**Woo 2012**[56]	Children, 8-16 years	167 (68)	> 22 ppb	57	87

Obesity is another confounding factor in the assessment of FeNO levels. A consistent body of data now indicates that asthma is closely related to obesity, and obese patients with asthma usually have more severe symptoms than non-obese asthmatics [66]. Indeed, obese asthma may be a unique phenotype that is characterized by more severe symptoms for a given degree of lung function impairment, destabilization or lack of asthma control, worse quality of life, lack of eosinophilic inflammation and a different response to controller medication [67]. At any age, obesity can adversely impact on lung function, and obesity duration is a determinant of lower pulmonary function [68]. FeNO levels have been extensively investigated in pediatric excess adiposity [9,69]. Some pediatric studies showed that FeNO is negatively associated with body mass index, waist-to-hip ratio, and percent body fat [70], while others found no relationship between FeNO levels and adiposity measures [69,71]. Furthermore, FeNO levels do not differ between obese and normal weight subjects with asthma [9]. A possible explanation for this results might be a selection bias, in particular overdiagnosis of asthma attributable to non specific obesity-related respiratory symptoms among obese children. Indeed, recent meta-analyses have pointed out that some obese patients with “asthma” may have respiratory symptoms caused by obesity without objective physiological criteria for asthma, or an exaggerated symptom perception [72].

As far as the discrimination between children with asthma and healthy subjects, FeNO measurement provides significantly higher diagnostic accuracy than lung function tests [58,73], and has diagnostic value comparable to that of conventional bronchial challenge tests [57]. Several studies showed an inverse correlation between FeNO levels and bronchial hyperreactivity tests in children [58,70-76], and only one group did not confirm this finding [77]. Nevertheless, elevated FeNO levels increase the probability of exercise-induced bronchoconstriction in asthmatic school-age children [78]. These conflicting results may be explained with the different methods used to measure FeNO as well as with the heterogeneity of the study populations, in particular regarding the presence of atopy and the use of steroids.

Cut-points rather than reference values have been proposed to interpret FeNO levels [33,34,72,79]. In children, FENO values less than 20 ppb indicate that eosinophilic inflammation is less likely, or that in patients presenting with non specific respiratory symptoms alternative diagnoses to asthma should be considered [79]. High FeNO concentration (>35 ppb in children) strongly suggests significant airway eosinophilia [79]. At high expiratory flows, ranging from 200 to 280 mL/s, the negative and positive predictive values for FeNO >25 ppb as predictor of asthma rise to 80% and 100%, respectively [80]. Nonetheless, intermediate FeNO levels (20-35 ppb in children) indicate that cautious interpretation in the etiology of the airway disorder is required.

An additional, novel, potential and attractive application of FeNO in asthma is the “prediction” of asthma onset. In the absence of symptoms, increased FeNO levels may reflect subclinical airway inflammation that may be predictive of “early asthma”, especially in allergic subjects [81,82]. This could be explained by an enhanced Th2 cytokine-driven airway response in allergic individuals that may precede the clinical presentation. Furthermore, in asymptomatic adolescents, increased FeNO may predict the development of rhinitis symptoms within a follow-up period of 4 years [83], suggesting that FeNO may be a sensitive biomarker of the “allergic march”. These findings have potential clinical and therapeutic implications, also because studies in animal models seem to show a possibility to block induction of Th2 responses, thus preventing the development of future asthma [84].

#### FeNO in the follow-up of bronchial asthma

The goal of asthma long-term treatment is to reduce inflammation for controlling symptoms. Treatment options are usually guided by symptoms and lung function. However, these factors do not reflect chronic airway inflammation. This is also shown by contrasting results of studies on the relationship between FeNO levels and both symptoms (including recent symptoms, symptom frequency, symptom scores), symptom control, or rescue ß2-agonist use [77,85-94] and pulmonary function test results [25,85-87,89,91,93-100].

Inhaled corticosteroids (ICS) are the first choice for asthma maintenance treatment. Interestingly, inhaled or systemic corticosteroid administration results in dose dependent reductions of FeNO levels [27,100]. Moreover, in corticosteroid-naïve patients with suspected asthma, the baseline FeNO value may predict an ICS response in terms of improved lung function and reduced airway reactivity [73]. Therefore, FeNO seems a suitable biomarker for modifying ICS dose in order to obtain better asthma control [101]. However, in children, daily monitoring of FeNO at home [102], as well as measurement of FeNO levels every 3 months for 1 year [103], or 5 times in 6 weeks [104] do not provide any advantage in improving the symptom score. In adolescents and adults, Szefler *et al* showed that the addition of FeNO measurement as an indicator of asthma control resulted in higher doses of ICS and long-acting β2 agonists than did standard guideline-based treatment, and did not determine improvements in asthma symptoms or lung function [105]. A recent meta-analysis concluded that the number of asthma exacerbations is not significantly reduced in adults and children when ICS was tailored based on FeNO [106].

These findings may be explained by the fact that day-to-day variations of FeNO are common and do not correlate with changes in symptom score [107]. Furthermore, some atopic asthmatic patients showed a lack of FeNO responsiveness to ICS [108,109], or may have increased FeNO levels despite high dose ICS [110].

Another issue is whether the change in FeNO values may be a better predictor than absolute levels. FeNO levels quickly decrease in response to ICS and therefore they may be useful to ascertain that ICS is regularly taken. Finally, baseline FeNO seems a worse predictor of asthma improvement than the change in FeNO after 80 days of ICS [111].

According to the Clinical Practice Guideline of the American Thoracic Society (ATS) [79] it has been suggested to consider as significant the increase in FeNO greater than 20% for values over 50 ppb, or more than 10 ppb for values lower than 50 ppb from one visit to the next. The second one recommends to use a reduction of at least 20% in FeNO for values over 50 ppb (or more than 10 ppb for values lower than 50 ppb) as the cut point to indicate as significant the response to antiinflammatory therapy.

The results of two recent studies indicate new possible clinical applications of FeNO measurement in pediatric asthma. Pifferi *et al* assessed the value of spirometry and FeNO measurements, alone or in combination, in models developed by a machine learning approach for the objective classification of asthma control [112]. The combined use of spirometry parameters and FeNO levels modeled by a soft computing learning approach applied to spirometry could discriminate the level of asthma control. Van der Valk *et al* found that FeNO measured daily by a hand-held device started to increase approximately 10 days before moderate exacerbations occurred, this suggesting that regular FeNO measurements in the home setting could help to detect and even to prevent the loss of asthma control [113]. Apart from ICS, other established controller therapies, such as leukotriene modifiers or anti-IgE therapy with omalizumab, have been demonstrated to reduce FeNO in children, alone or combined with ICS [114-117].

On the basis of the studies that have provided evidence regarding the applications of NO measurements in clinical practice, ATS recently indicated the rationale for FeNO measurement in asthma, even in the pediatric population [79], highlighting the following situations:

– Diagnosis of eosinophilic airway inflammation

– Support of asthma diagnosis when objective evidence is needed

– Baseline evaluation and follow-up monitoring of airway inflammation

– Assessment of potential response or failure to respond to inhaled corticosteroids

– Evaluation of adherence to antiinflammatory medications

– Guide for dose changes in antiinflammatory medications

### FeNO in viral bronchiolitis

Respiratory Syncytial Virus (RSV) manifests restricted tropism for the respiratory epithelium stimulating an inflammatory response [118]. RSV increases NOS2 messenger RNA, and upregulates NOS2 and its nitrite products in lines and cultures of respiratory epithelial cells [119-121]. Despite in vitro and animal data demonstrated NO involvement in bronchiolitis [120,122], only few studies evaluated FeNO levels in children with RSV bronchiolitis. Surprisingly, FeNO appeared significantly lower in infants with bronchiolitis than in healthy controls or in preschool children with recurrent wheezing [123]. However, the same study demonstrated that 3 months after the diagnosis of RSV bronchiolitis, FeNO appeared significantly higher in affected children than in normal subjects, suggesting that low FeNO reflects the active suppression of NO production occurring during the active infection, while the increased levels might be interpreted as a “rebound” phenomenon. Future studies will hopefully provide more insights on the relationship between viral infections and subsequent chronic bronchial asthma.

### FeNO in community acquired pneumonia

The scientific literature on FeNO modifications during community acquired pneumonia (CAP) in children is scarce. Since NO is part of the innate inflammatory response, its levels are expected to rise in acute lung infection. Healthy children with an abnormally high FeNO had significantly increased frequency of previous bronchitis or pneumonia in the past year [124]. However, Carraro and colleagues measured FeNO three times over a 1 month period in the exhaled breath condensate obtained from children with chest x-ray evidence of CAP, and found no significant differences in FeNO levels either from children with CAP compared to healthy controls, or from baseline levels during the follow-up [125]. Different hypotheses might explain this paradox. First, NO output from expiratory flows of 50 mL/s mainly derives from airway NO diffusion, and therefore at higher flow rates, that sample from the deeper parts of the lung, higher NO levels might be found. Second, NO might also react rapidly with reactive oxygen species, forming NO-metabolites. Unfortunately, the above mentioned data do not allow to reach definitive conclusions, and highlight the need for further studies on the role of FeNO in children with CAP.

### FeNO in bronchiectasis

Bronchiectasis is caused by, or associated with, many disorders including congenital/genetic conditions, e.g. cystic fibrosis (CF), primary immunodeficiency, primary ciliary dyskinesia (PCD), Mounier-Kuhn syndrome, chronic obstructive pulmonary disease, bronchiolitis obliterans, sarcoidosis, autoimmune disorders, and acquired post-infectious diseases [126-131]. High-resolution computed tomography (HRCT) is the gold standard for the diagnosis [128,132], even though radiation, magnetic resonance imaging has been proposed as an alternative radiation-free technique, especially in children [133,134].

Levels of inflammation in stable bronchiectasis seem to correlate with a reduction of patient’s quality of life [135]. Therefore, monitoring inflammation is of outstanding relevance in the management of affected patients, especially for preventing the disease progression. Since current markers of inflammation in the blood and in the sputum are indirect, variable and invasive, FeNO measurement may represent a useful way to assess airway inflammation in patients with bronchiectasis. Some studies found that FeNO levels from adults with non-CF, non-PCD bronchiectasis are significantly higher compared to controls [136-138], but others did not confirm this [80,139,140]. In children with bronchiectasis due to CF or PCD, FeNO levels are abnormally low compared to non CF- non PCD-patients or to controls, but there are no significant differences between PCD and CF [80,141-143].

NO measurement at different expiratory flow rates allows to assess the contribution of NO from different parts of the lung. A two-compartment model of pulmonary NO exchange dynamics has been proposed to demonstrate relative contributions of bronchial (J) and peripheral (Calv) airway NO to the final FeNO concentration [144]. In adults with non CF- non PCD- bronchiectasis an increase in CalvNO with normal JNO levels has been recently demonstrated [136]. In children with CF-related bronchiectasis results appear conflicting, with one study showing lower JNO in CF than controls and no difference in CalvNO between groups [145], whereas other authors demonstrated that children with CF had a significantly higher CalvNO, but no significant difference in JNO compared to healthy children [146]. On the other hand, in children with PCD, very low levels of nasal NO are still a partially unexplained feature, whereas FeNO levels show considerable overlap with healthy subjects [80]. Paraskakis and colleagues found that in children with PCD JNO was significantly reduced, but CalvNO was normal compared with healthy controls [147]. On the basis of these findings, authors speculate that the normal CalvNO values militate against a generalized disorder of NO metabolism in PCD children, and hypothesize that NOS3 (endothelial) has normal function in PCD, while the uncoupling of the contractile process of the cilia from NOS2 (inducible) may result in failure of NO production.

Notwithstanding the conflicting results between affected children and adults, FeNO measurement could represent a useful non invasive tool to monitor bronchial inflammation over time.

### FeNO in diffuse lung disease

The term diffuse lung disease (DLD) encompasses a heterogeneous group of chronic respiratory disorders characterized by abnormal gas exchange and diffuse radiographic and histopathologic abnormalities [148]. DLD is rare in children, and diagnosis requires detailed history, complete physical examination, lung imaging, pulmonary function testing, bronchoalveolar lavage (BAL), and in most cases an open lung biopsy to confirm the suspicion [148,149].

Several studies evaluated FeNO levels in adults with DLD. In systemic sclerosis, FeNO concentrations are lower in subjects with interstitial lung involvement than in those without, whereas patients without pulmonary disease have higher FeNO than healthy subjects [150]. Moreover, FeNO levels seem to be increased in subjects with asbestosis [151], but not in pulmonary sarcoidosis [152]. To the best of our knowledge, no studies ever evaluated FeNO levels in children with DLD. This would likely be crucial in the management of these critical conditions, hopefully being useful in the evaluation of the progression of the disease, or of the response to treatment.

### FeNO in bronchiolitis obliterans

Bronchiolitis obliterans (BO) is almost secondary to lung transplantation in adults [153], while the most common form in children is post-infectious BO [154]. FeNO levels are increased in adult BO that occurs after lung transplantation [155,156], and correlate with NOS2 expression in the bronchial epithelium and with the percentage of BAL neutrophils [157-161]. No pediatric studies evaluating the role of FeNO in children with BO after lung transplantation have been published. The availability of a non-invasive biomarker that can identify patients in whom more invasive diagnostic procedures such as BAL or lung biopsies are justified (or can be avoided) would be very helpful to improve the management of children with BO.

## Conclusions

NO seems to significantly influence a variety of physiological and pathophysiological processes in the upper and lower airways. FeNO measurement is easy to perform, and seems reproducible also in the preschool age. The availability of hand-held devices will hopefully increase its use in the pediatric practice. Combined with symptoms registration and lung function measurements, FeNO provides additional information that can be applied to support the diagnosis of asthma and to optimize the management of affected children. Moreover, recent studies suggest that FeNO is helpful in predicting both onset and exacerbations of asthma. Since most studies have been performed in adult populations, more research is needed to confirm the usefulness of NO measurement in the diagnosis and management of pediatric chronic airway disorders different from asthma.

## Abbreviations

NO: Nitric oxide; NOS: NO synthase; FeNO: Fractional exhaled nitric oxide; ICS: Inhaled corticosteroids; RSV: Respiratory syncytial virus; CAP: Community acquired pneumonia; CF: Cystic fibrosis, PCD, Primary ciliary dyskinesia; HRCT: High-resolution computed tomography; DLD: Diffuse lung disease; BAL: Bronchoalveolar lavage; BO: Bronchiolitis obliterans.

## Competing interests

All authors declare that they have no significant competing financial, professional or personal interests that might have influenced the performance or presentation of the work described in this manuscript.

## Authors’ contributions

AM has been involved in drafting the manuscript; CC has been involved in revising the manuscript critically for important intellectual content; MV has made substantial contributions to acquisition, analysis and interpretation of data; CP has made substantial contributions to acquisition, analysis and interpretation of data; SM has been involved in drafting the manuscript; MM has been involved in drafting the manuscript; FSp has made substantial contributions to acquisition, analysis and interpretation of data; FS has been involved in drafting the manuscript, revising it critically for important intellectual content and has given final approval of the version to be published. All authors read and approved the final manuscript.
